# Supplementary material for: miR-450-5p and miR-202-5p Synergistically Regulate Follicle Development in Black Goat
Source: Int J Mol Sci. 2022 Dec 26;24(1):401. doi: 10.3390/ijms24010401 (PMC9820456; doi:10.3390/ijms24010401)
Supplement: Supplementary file 1 [file ijms-24-00401-s001.zip › Supplementary material.pdf]

Supplementary material

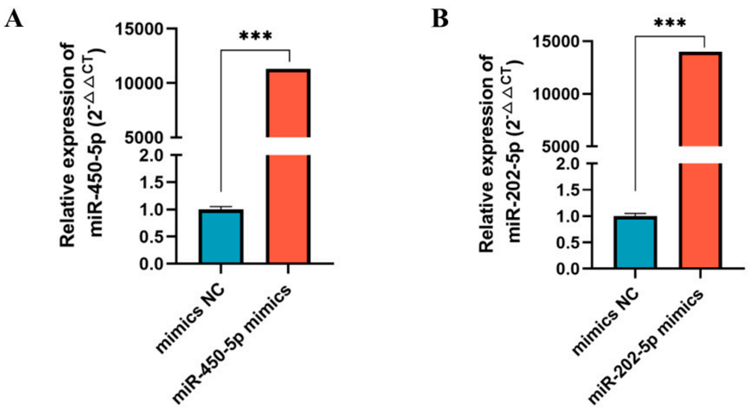

Supplementary Figure S1. Transfection efficiency validation (A) Expression of overexpressed miR-450-5p. (B) Expression of overexpressed miR-202-5p.

Supplementary Table S1. Statistics of the mapping result

Sample	Total_Mapped	Multiple_Mapped	Uniquely_Mapped
1LF	91691321 (87.83%)	3985537 (4.35%)	87705784 (95.65%)
2LF	91201935 (90.11%)	3364788 (3.69%)	87837147 (96.31%)
3LF	91849800 (90.64%)	4136097 (4.50%)	87713703 (95.50%)
4LF	91302301 (90.12%)	2927907 (3.21%)	88374394 (96.79%)
5LF	92564834 (91.66%)	3744245 (4.04%)	88820589 (95.96%)
1SF	91353989 (86.60%)	2626005 (2.87%)	88727984 (97.13%)
2SF	92006429 (88.17%)	4035230 (4.39%)	87971199 (95.61%)
3SF	88991968 (87.52%)	3132153 (3.52%)	85859815 (96.48%)
4SF	89492103 (84.84%)	2812224 (3.14%)	86679879 (96.86%)
5SF	87490498 (82.56%)	2911392 (3.33%)	84579106 (96.67%)

Multiple-Mapped, the total number of sequences aligned to multiple positions; The uniquely mapped number of sequences with unique alignment positions on the reference sequence

Supplementary Table S2. Real-time fluorescence quantification primer list

Name	Type	F/R	Sequence(5′-3')	Product size	Reference
TRIM7	mRNA	F	AAGGTAGGGGCAGAGTTCCA	243	XM_018050549.1
		R	CACATTGCTGCACCTGCTTA		
PDK4	mRNA	F	TGGTGTTCCCCTGAGAGTCA	109	XM_005678949.3
		R	GTAACCAAAACCAGCCAGCG		
BANK1	mRNA	F	CCCACAAACATCCCTCCGAA	131	XM_018049269.1
		R	TCCATCCGACCTCTGTCTTG		
CAMK2N1	mRNA	F	CGGAGCAAGCGGGTTGTTAT	205	

		R	GCGGGTAGCAAGCTAGTGAT		XM_018055010.1
DSG	mRNA	F	GGATTGGTCCCGTTCTTGCT	151	XM_018039432.1
		R	TATTGGTCAGAGCCCCAGGA		
ELMO1	mRNA	F	TAGAGGCAGAGCCACTCACA	149	XM_018047159.1
		R	CACGTGTCTATACCTGCCCG		
HCK	mRNA	F	GCCCACTATTCGAAGGGGAG	193	XM_018057690.1
		R	CCACCTTGGTGTGCTTGTTG		
INPP5D	mRNA	F	ACCGCTTGTTCTATAACCTTG	211	XM_018041075.1
		R	CCCTGTAACCCTGTCACTCC		
LCP	mRNA	F	GATGCTACCCTTTCACAGA	216	XM_005687438.3
		R	TCAGACCTCCACGCTTAC		
MS4A130	mRNA	F	TCCAGACCCAAAGCTAACGTC	300	XM_005690341.3
		R	GTCTCTGGGGAGCTGTGAAC		
BMF	mRNA	F	CAGACTCTCAGCCCAGCTTC	258	XM_018054467.1
		R	TCGGTTCTGCTGATGTTGCT		
BMF	mRNA	F	CCCTCCTTCCCAATCGAGTC	133	NM_001003940.2
		R	GATGGCTCCATCTCTCCTGG		
BCL2	mRNA	F	GGATAACGGAGGCTGGGATG	156	NM_000633.3
		R	TGACTTCACTTGTGGCCCAG		
ENSCHIG00000001184	lncRNA	F	AAGCACCCAAACTGTCCA	146	
		R	TCCCGTGAAACCCCGT		
ENSCHIG00000002042	lncRNA	F	CTTGTTGTTTACCTGTTCG	92	
		R	TGGTTGGCTGATGTTCT		
ENSCHIG00000000794	lncRNA	F	CAAAGCAGTGTTGGTCC	140	
		R	TGGTAAAGAATCCGCC		
ENSCHIG00000003632	lncRNA	F	GCTGCTATCATTACCCTCT	85	
		R	CTTGTTCCCTTTCCTCC		
ENSCHIG00000004556	lncRNA	F	GCGGATTCTTTACCCTC	80	
		R	TCTTCCTTTGTGCCAGT		
ACTIN	mRNA	F	CCTGCGGCATTCACGAAAC	187	NM_001314342.1
		R	GGGGGCGCGATGATCTTGA		

Supplementary Table S3.RNA oligonucleotides sequence information

Sequence name	Fragment sequence (5'-3')	Application

miR-450-5p mimics	UUUUGCGAUGUGUUCCUAAU	miRNA overexpression
miR-202-5p mimics	UUCCUAUGCAUAUACUUCUUU	miRNA overexpression
si-BMF	GTGTACATACAACATACTCAAGC	BMFknockdown
