# Supplementary figures and images for: miR-450-5p and miR-202-5p Synergistically Regulate Follicle Development in Black Goat
Source: Int J Mol Sci. 2022 Dec 26;24(1):401. doi: 10.3390/ijms24010401 (PMC9820456; doi:10.3390/ijms24010401)

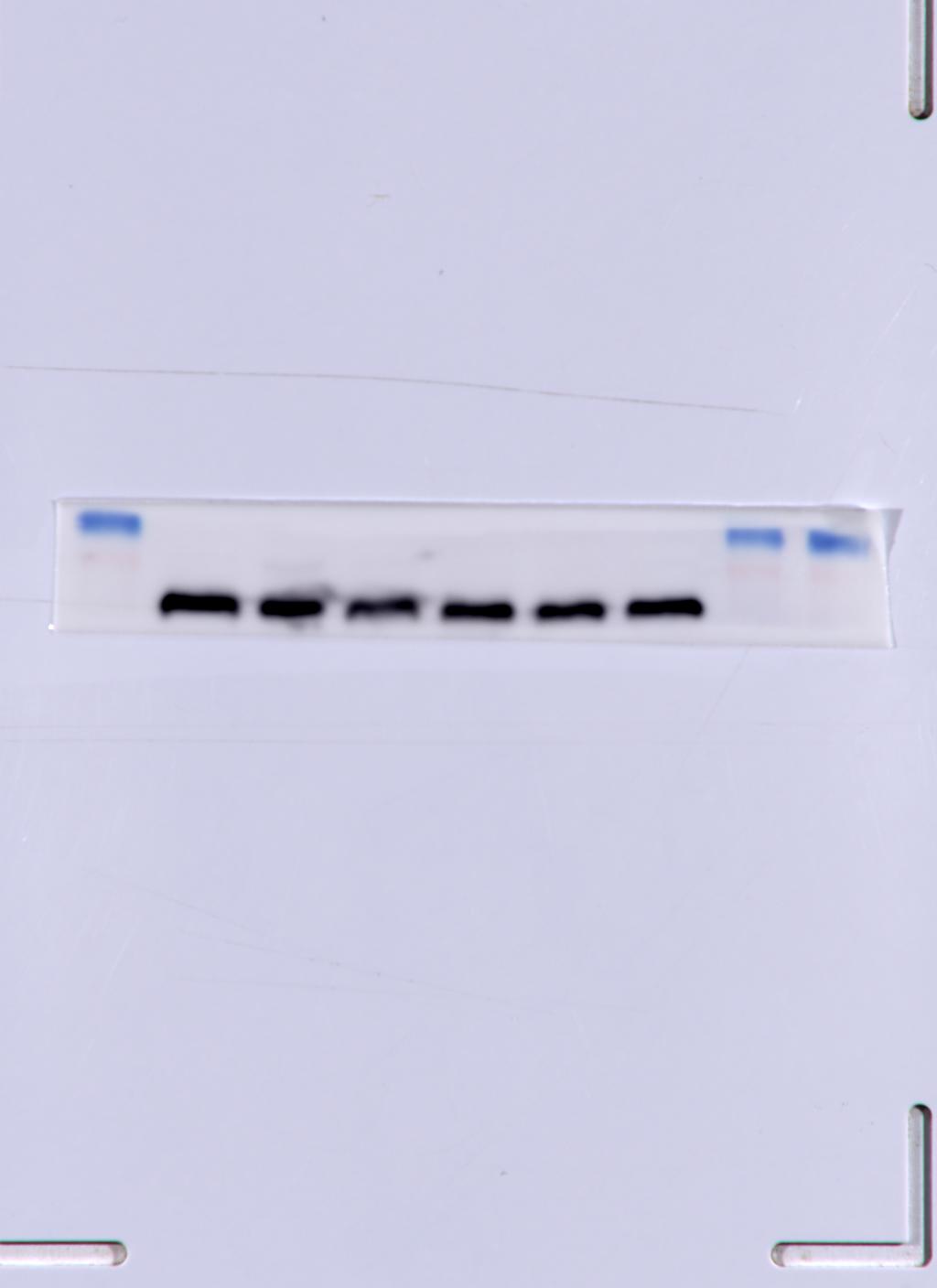

Supplement: Supplementary file 1 [file ijms-24-00401-s001.zip › Supplementary information for western blot/Supplementary Information on Western blot results after knockdown of BMF/siBMF Actin.jpg]

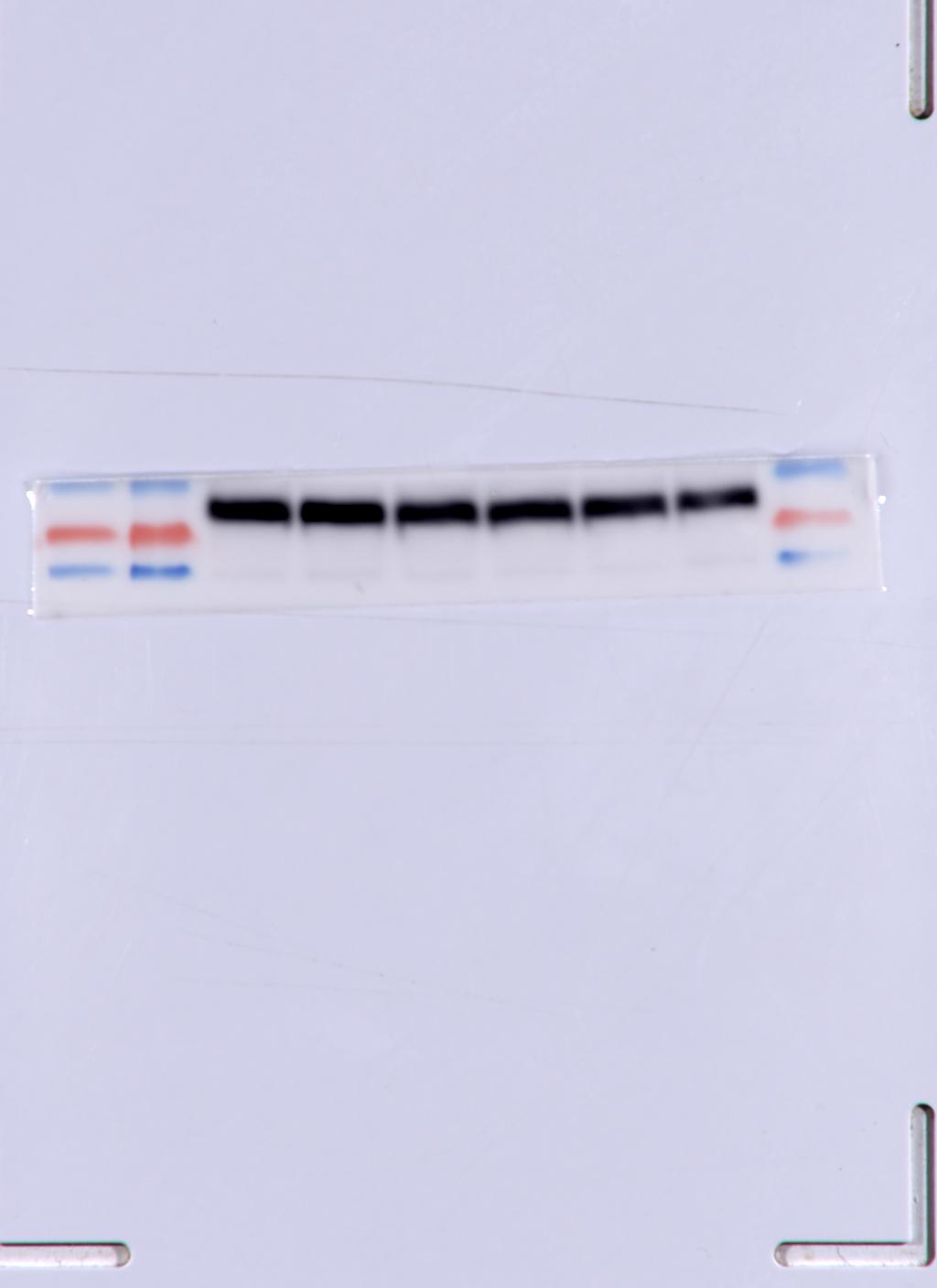

Supplement: Supplementary file 1 [file ijms-24-00401-s001.zip › Supplementary information for western blot/Supplementary Information on Western blot results after knockdown of BMF/siBMF AKT.jpg]

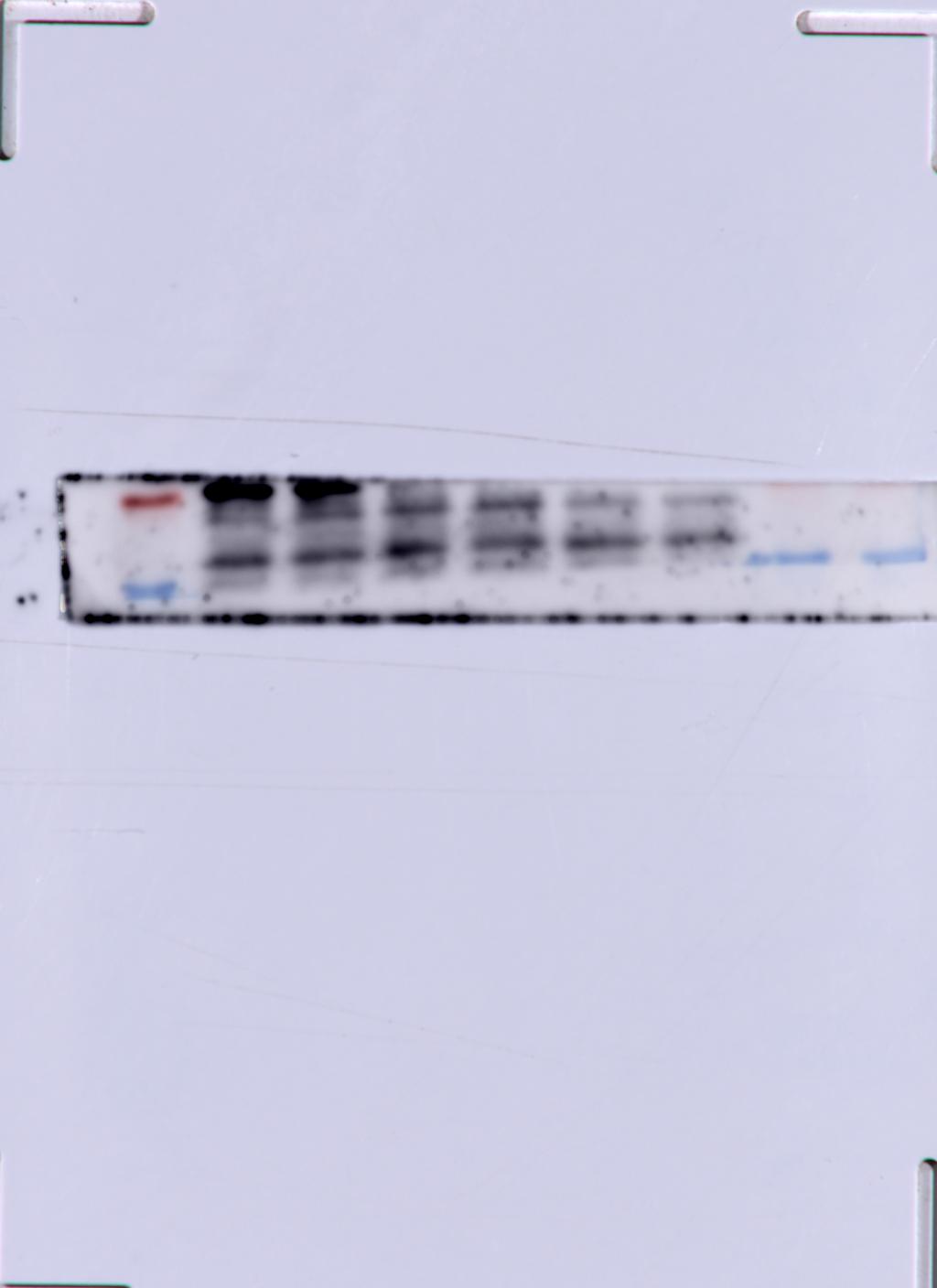

Supplement: Supplementary file 1 [file ijms-24-00401-s001.zip › Supplementary information for western blot/Supplementary Information on Western blot results after knockdown of BMF/siBMF BMF.jpg]

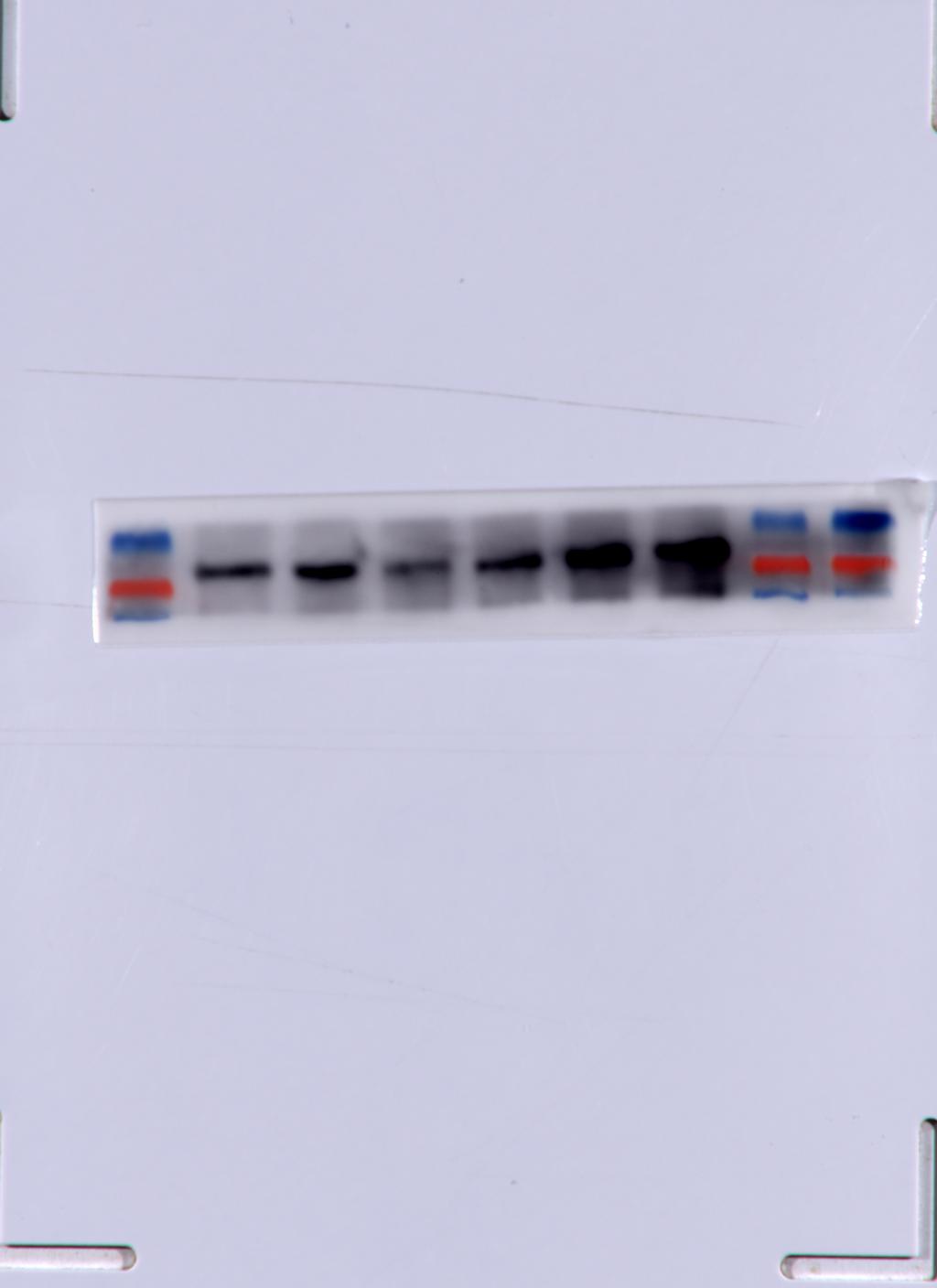

Supplement: Supplementary file 1 [file ijms-24-00401-s001.zip › Supplementary information for western blot/Supplementary Information on Western blot results after knockdown of BMF/siBMF p-AKT.jpg]

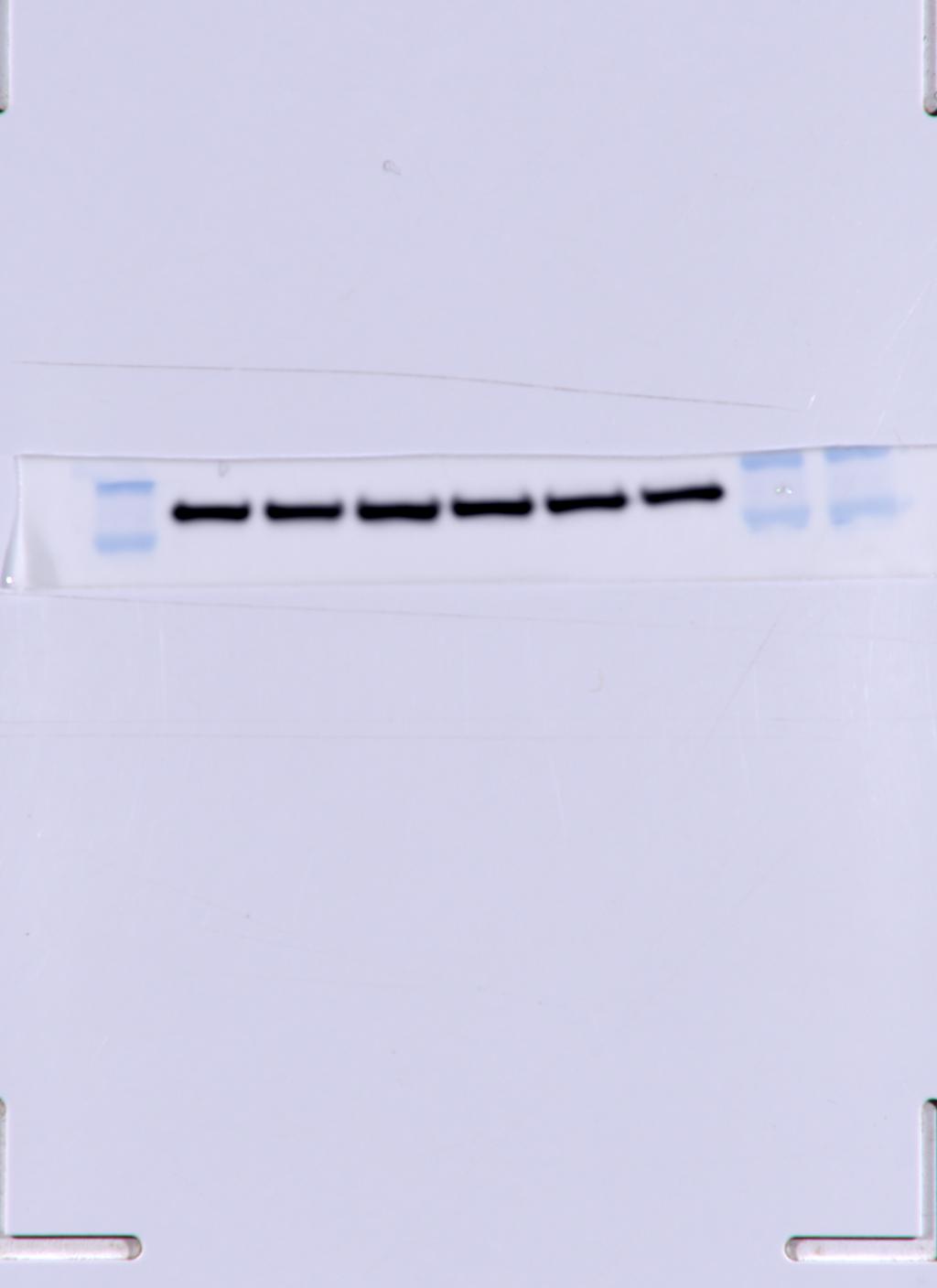

Supplement: Supplementary file 1 [file ijms-24-00401-s001.zip › Supplementary information for western blot/Supplementary information on Western blot results after overexpression of miR-202-5p/Actin.jpg]

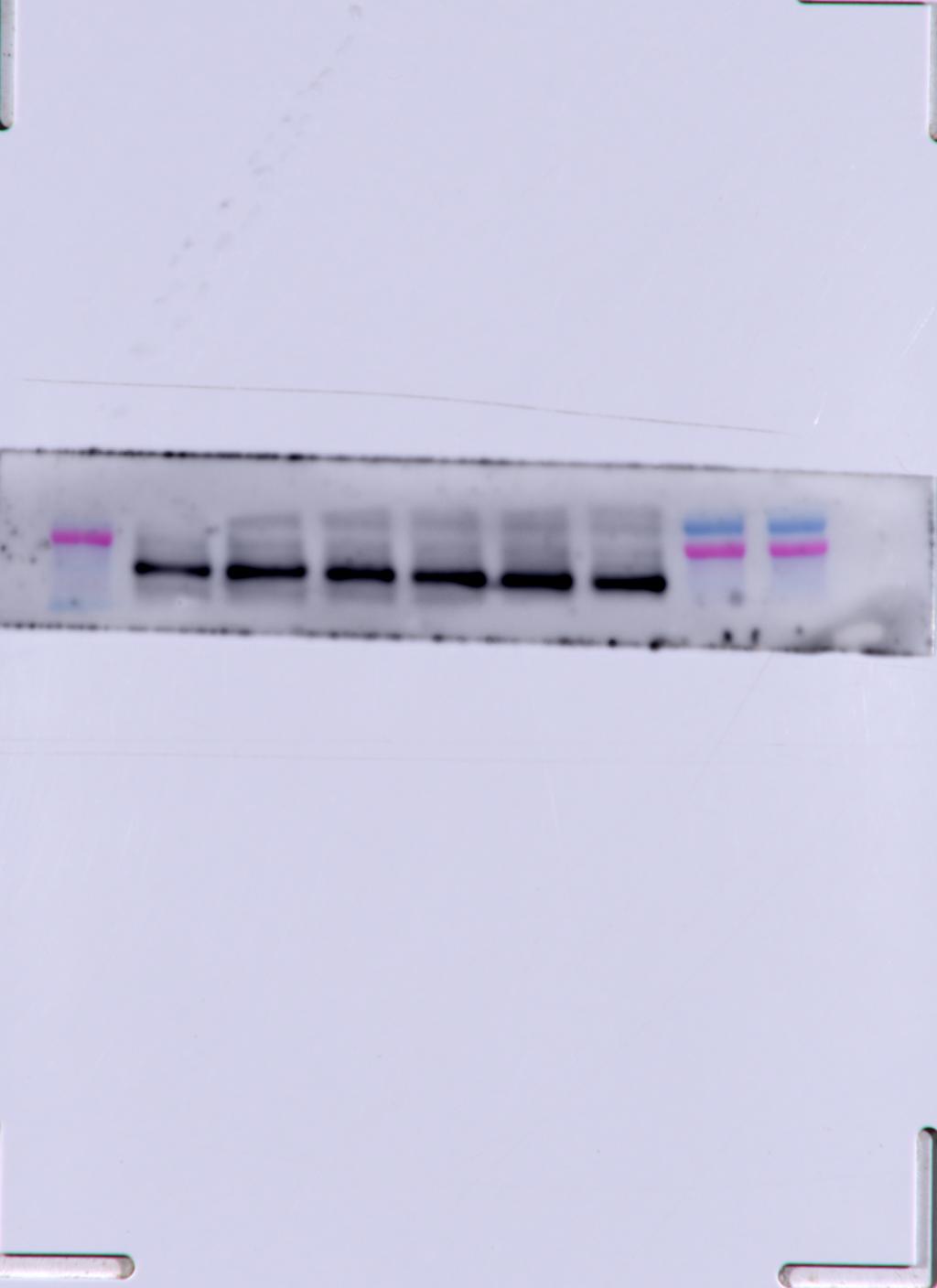

Supplement: Supplementary file 1 [file ijms-24-00401-s001.zip › Supplementary information for western blot/Supplementary information on Western blot results after overexpression of miR-202-5p/AMPK.jpg]

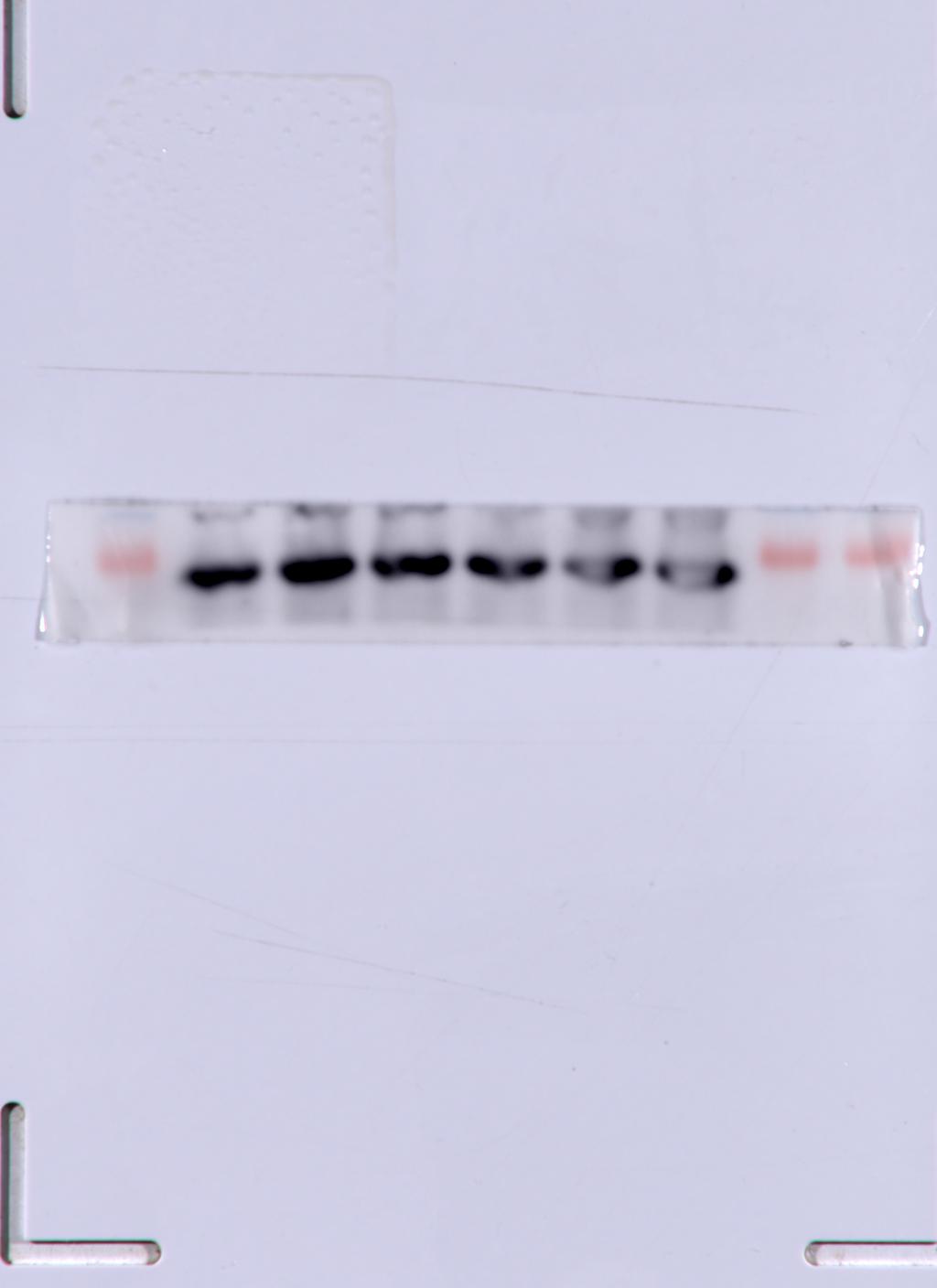

Supplement: Supplementary file 1 [file ijms-24-00401-s001.zip › Supplementary information for western blot/Supplementary information on Western blot results after overexpression of miR-202-5p/BCL2.jpg]

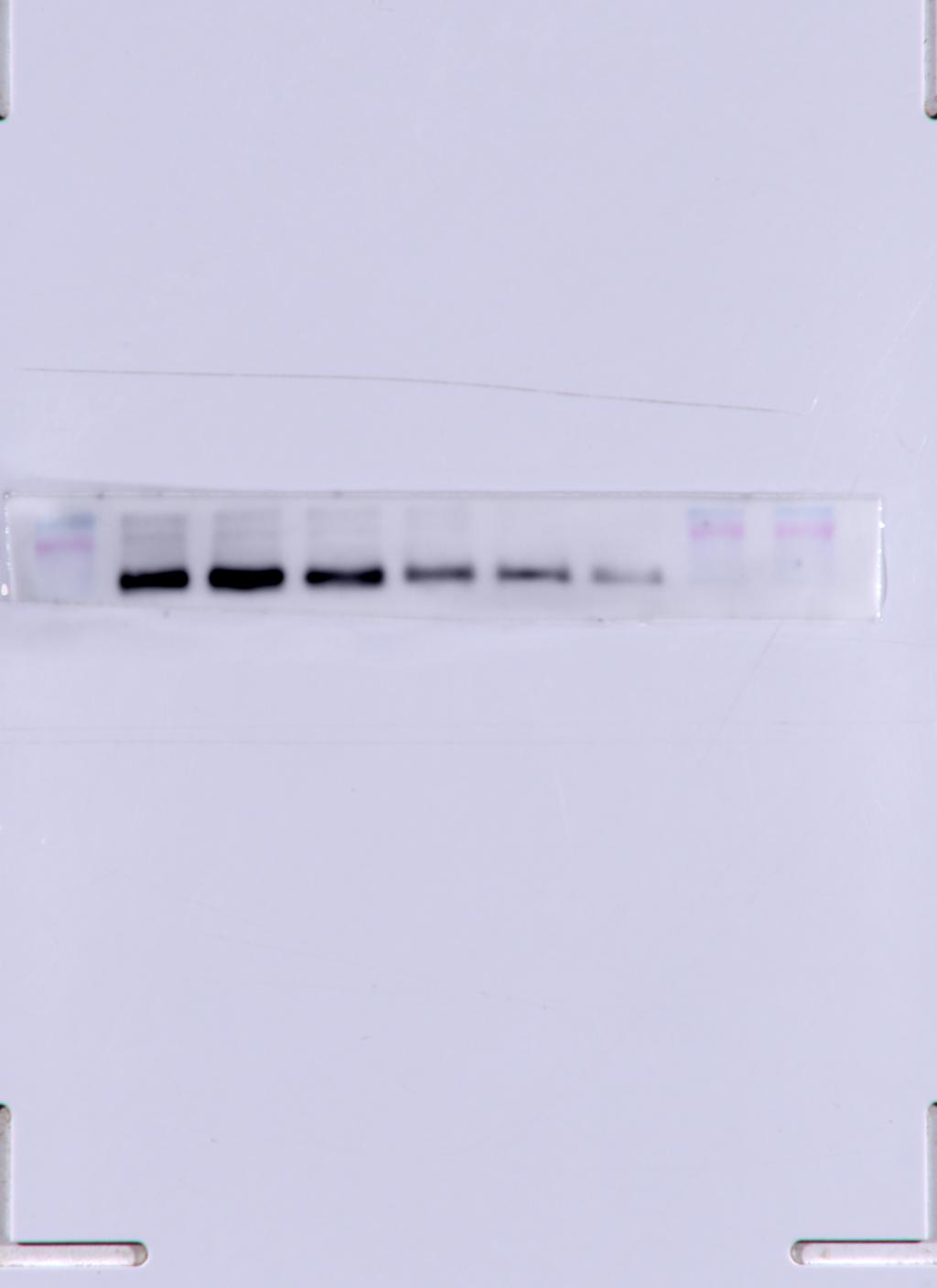

Supplement: Supplementary file 1 [file ijms-24-00401-s001.zip › Supplementary information for western blot/Supplementary information on Western blot results after overexpression of miR-202-5p/P-AMPK.jpg]

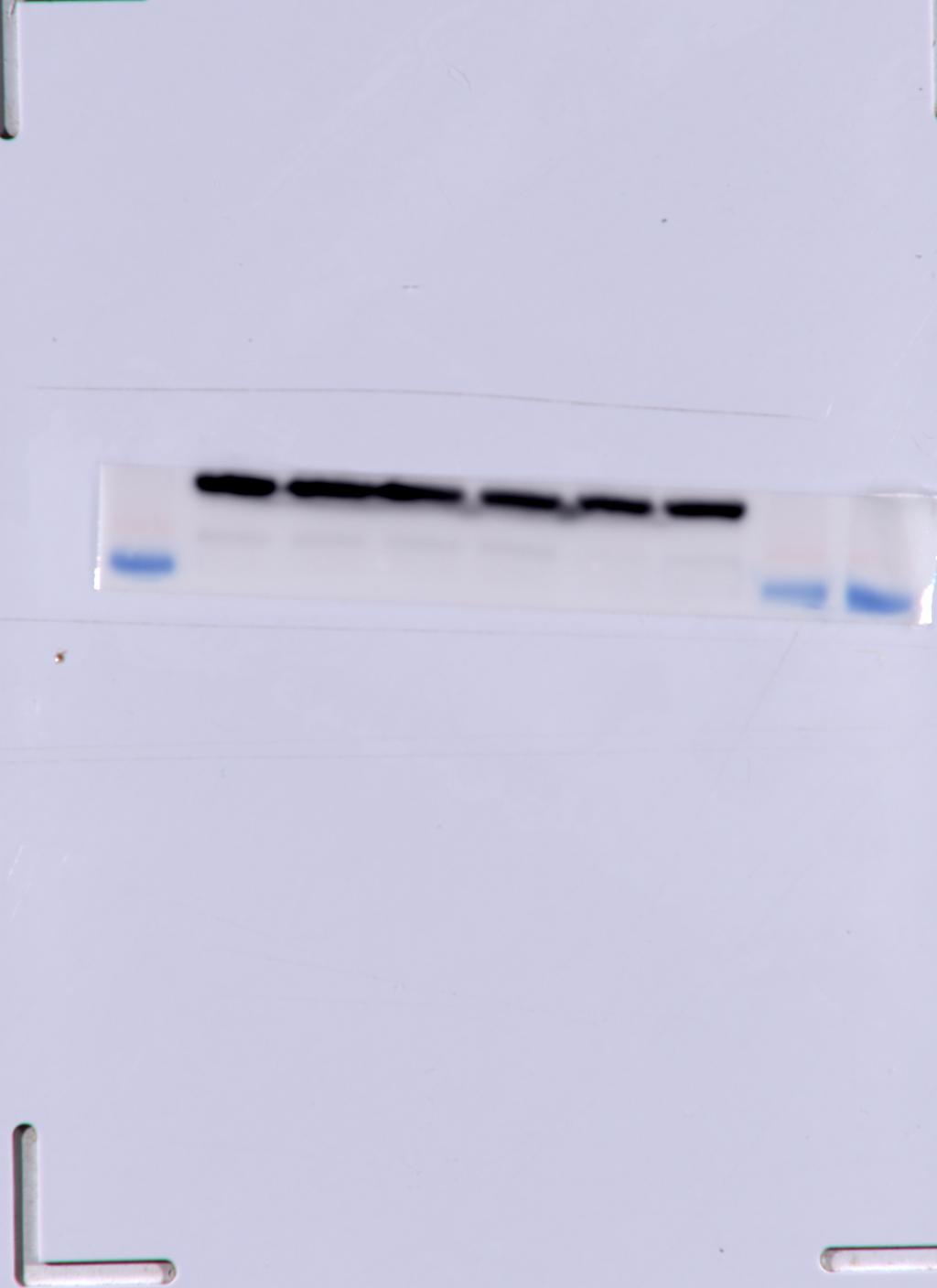

Supplement: Supplementary file 1 [file ijms-24-00401-s001.zip › Supplementary information for western blot/Supplementary information on Western blot results after overexpression of miR-450-5p/Actin.jpg]

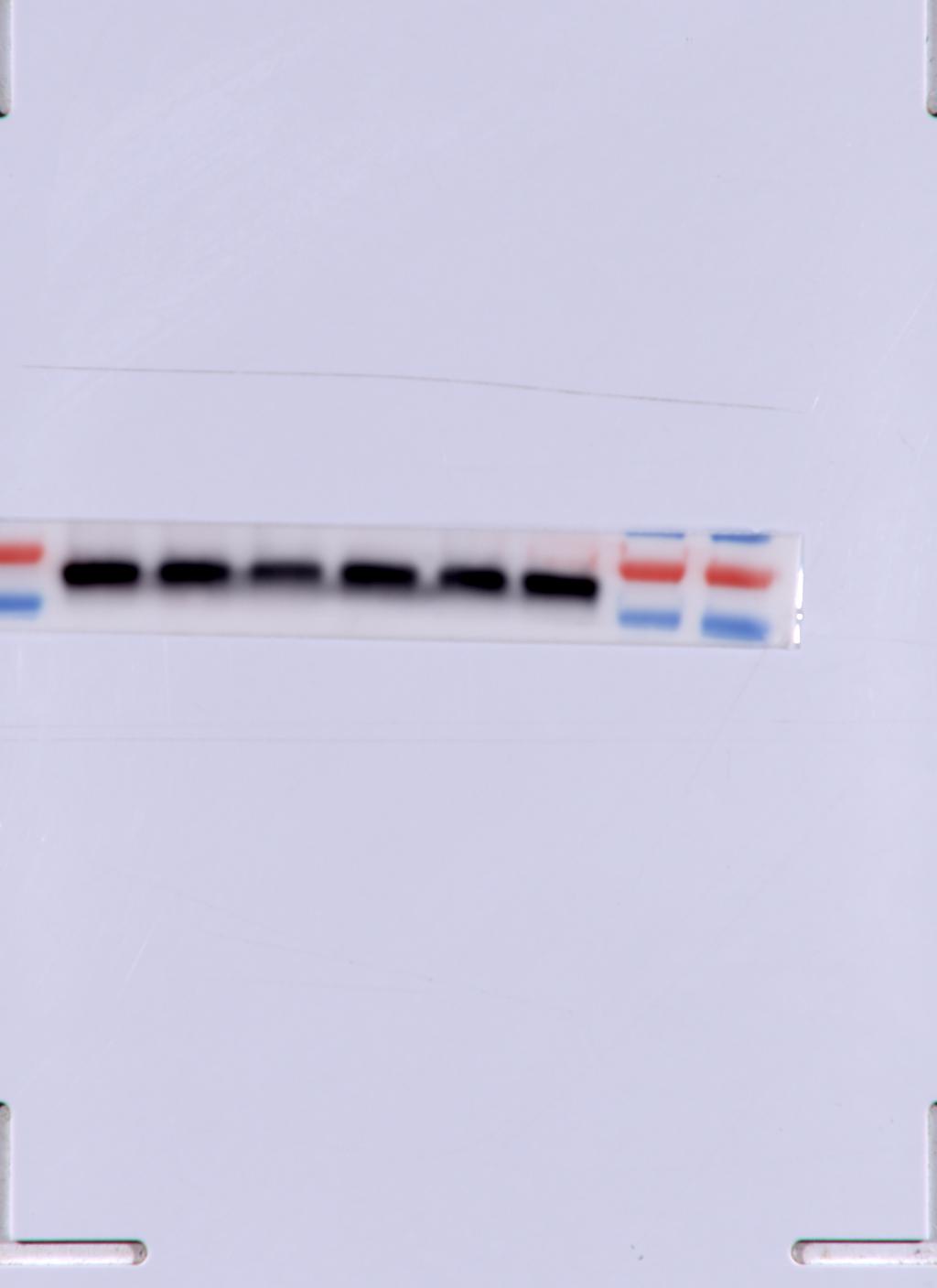

Supplement: Supplementary file 1 [file ijms-24-00401-s001.zip › Supplementary information for western blot/Supplementary information on Western blot results after overexpression of miR-450-5p/AKT.jpg]

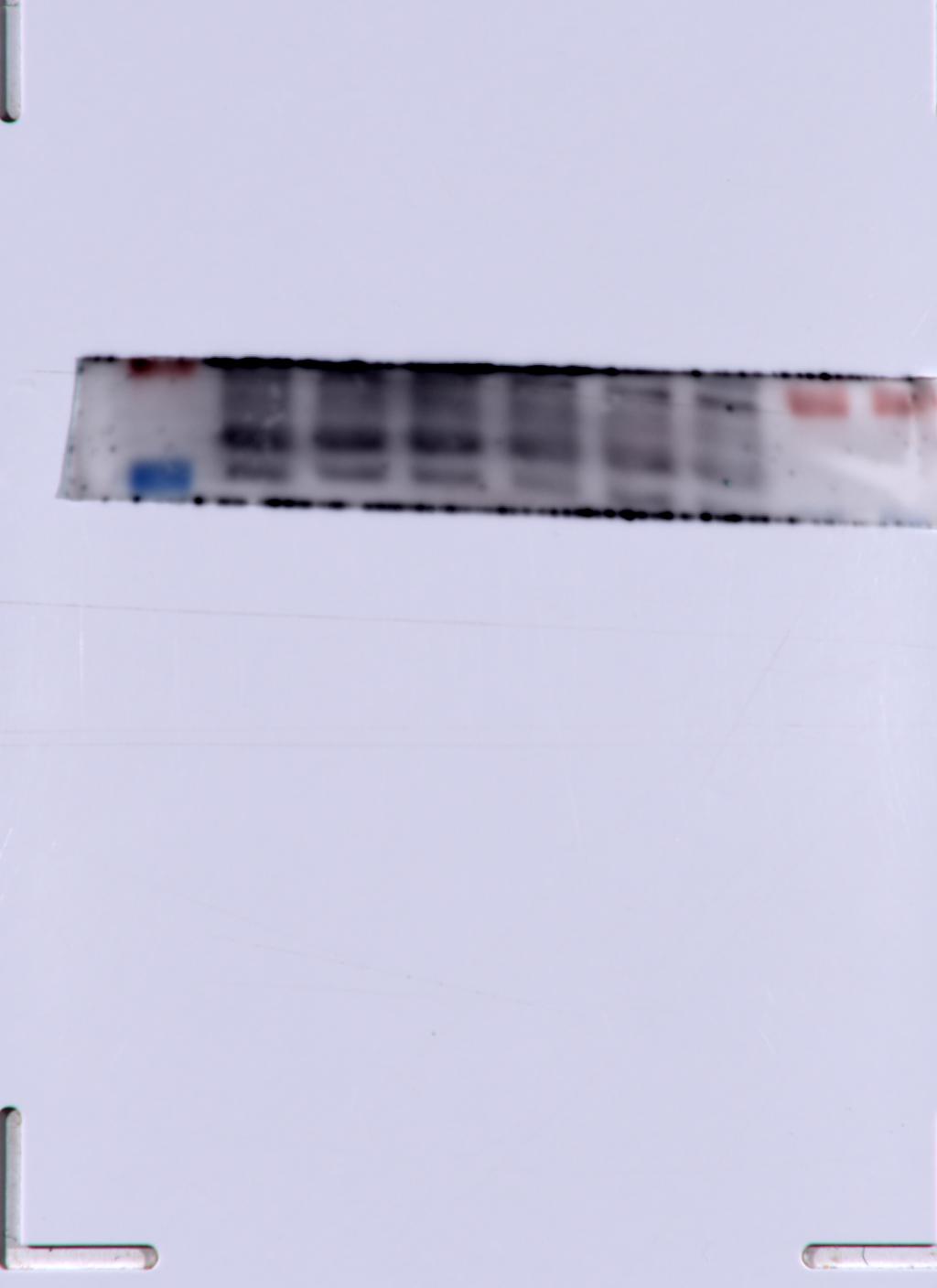

Supplement: Supplementary file 1 [file ijms-24-00401-s001.zip › Supplementary information for western blot/Supplementary information on Western blot results after overexpression of miR-450-5p/BMF.jpg]

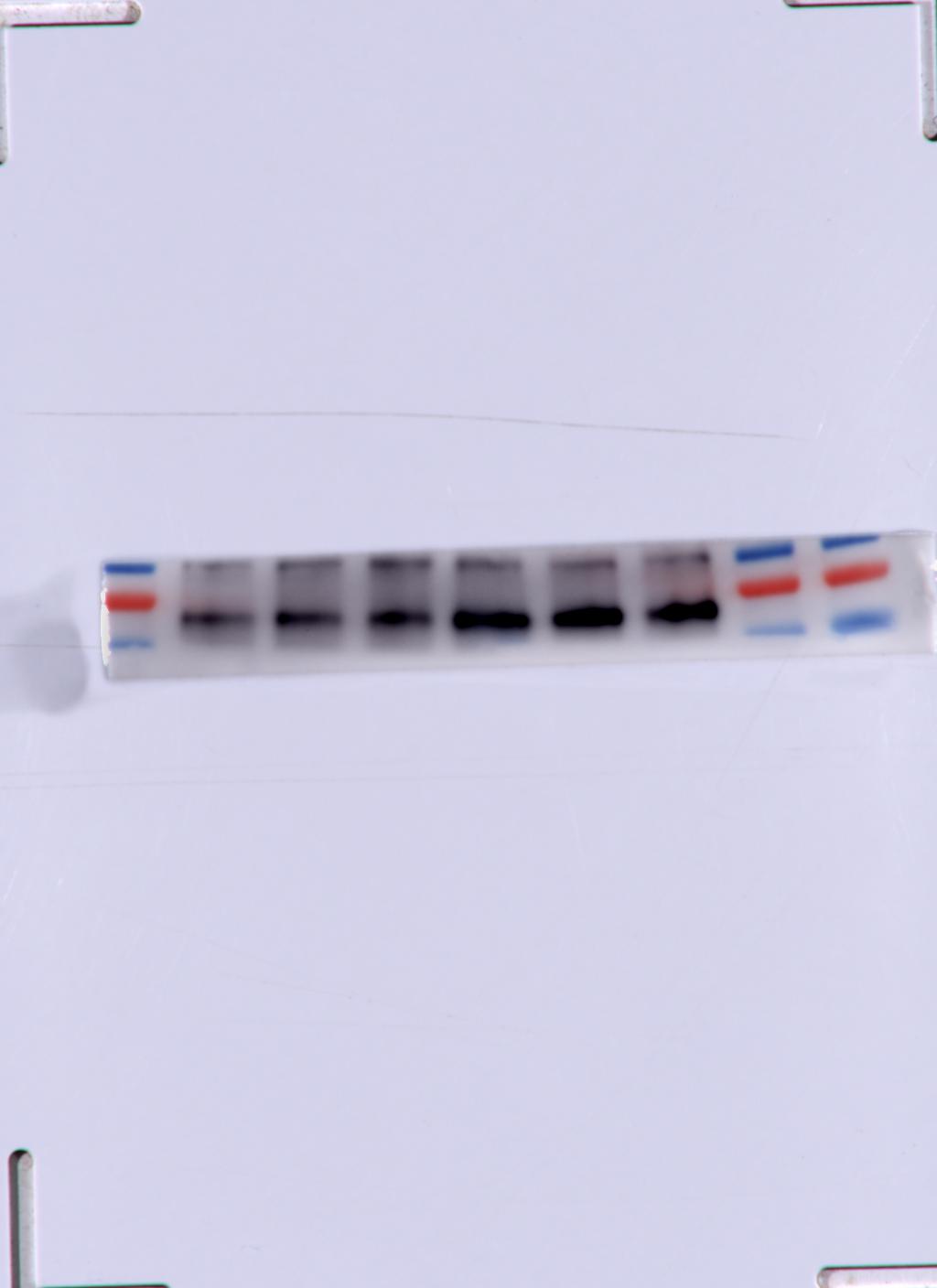

Supplement: Supplementary file 1 [file ijms-24-00401-s001.zip › Supplementary information for western blot/Supplementary information on Western blot results after overexpression of miR-450-5p/p-AKT.jpg]
